# Mixed channel traffic grooming with crosstalk awareness and shared backup path protection in IP over multicore fibre elastic optical networks

**DOI:** 10.1038/s41598-026-55987-8

**Published:** 2026-06-05

**Authors:** Fengxian Tang, Yuan Zheng, Yongxue Wang, Shifeng Ding

**Affiliations:** 1 School of Electronic and Communication Engineering, Shenzhen Polytechnic University, Shenzhen, 518000 Guangdong People’s Republic of China; 2 School of Electronic and Information Engineering, Suzhou University of Science and Technology, Suzhou, 215000 Jiangsu People’s Republic of China

**Keywords:** Elastic optical networks, Multicore fibre, Inter-core crosstalk, Traffic grooming, Shared backup path protection, IP-over-optical networks, Engineering, Mathematics and computing

## Abstract

Multicore fibre elastic optical networks (MCF-EONs) offer a promising route to overcome the capacity limit of single-core systems, but practical deployment is hindered by inter-core crosstalk and by the poor resource efficiency of dedicated protection. Here we address mixed channel traffic grooming in IP over MCF-EONs protected by shared backup paths, in which working and protection flows can be aggregated onto common optical channels subject to crosstalk constraints. We develop a double auxiliary-graph routing, core and spectrum assignment framework (Double-AG-RCSA) that jointly coordinates IP-layer grooming with crosstalk aware optical-layer provisioning. Simulations on two backbone topologies equipped with 7-core and 19-core fibres show that the proposed framework reduces the maximum used frequency-slot index by up to 33%, lowers the average inter-core crosstalk by about 6 dB, and decreases transponder usage by 28 to 35% relative to representative baselines, while eliminating crosstalk-threshold violations. The results demonstrate that cross-layer coordination of grooming, crosstalk aware provisioning and shared backup protection improves survivability, spectrum efficiency and signal quality in multi-layer optical networks. The framework is compatible with existing control planes and can be extended to dynamic traffic and larger topologies.

**Supplementary Information:**

The online version contains supplementary material available at 10.1038/s41598-026-55987-8.

## Introduction

The continuous growth of Internet traffic, driven by cloud computing, artificial intelligence and high-definition video, has pushed optical transport networks close to their capacity limits. Elastic optical networks (EONs) were introduced to improve spectral efficiency through flexible bandwidth allocation^1^, yet the transmission capacity of single-core fibres (SCFs) is approaching its fundamental limit^2^. Space-division multiplexing (SDM) has been proposed as a promising response^3^, and multicore fibre (MCF) technology is widely regarded as the most practical implementation because of its technological maturity and compatibility with existing infrastructure^4^.

Deploying MCF creates two new design pressures. First, most IP-layer demands do not occupy an entire spatial or spectral channel, so traffic grooming (TG) at the IP layer is used to aggregate sub-wavelength flows onto high-capacity optical channels, improving spectrum utilisation and reducing transponder counts^5,6^. Grooming, however, tends to place connections in neighbouring cores or adjacent spectrum slots; when multiple channels are co-located, inter-core crosstalk (XT) accumulates with transmission distance and can severely degrade the signal^7–9^. XT therefore becomes a key constraint on grooming and network planning. Second, survivability is essential in large-scale EONs, where fibre cuts or equipment faults must be restored quickly to meet service-level agreements. Dedicated 1 + 1 protection is simple and fast but wastes capacity; shared backup path protection (SBPP) allows a single backup resource to protect multiple mutually link-disjoint working paths and thereby significantly reduces reserved capacity, improves utilisation and lowers blocking^10,11^. In MCF-EONs, SBPP must additionally account for XT and core-assignment constraints, because placing working and protection channels in adjacent cores affects reachability and quality of transmission.

Previous work has addressed these problems largely in isolation. XT-aware RCSA schemes for MCF-EONs range from integer-linear-programming formulations^7^ and guard-core or counter-propagating assignments^8,12^ to machine-learning-based strategies^13,14^. Traffic-grooming studies have improved bandwidth utilisation through multi-layer coordination and learning-assisted prediction^15–18^, but predominantly in single-core settings. Protection studies have designed SBPP heuristics with a few extensions incorporating XT awareness^19–22^. Meanwhile, emerging bandwidth-intensive applications — from UAV-based sensing networks^23^ and privacy-preserving federated computing^24^ to large-scale health-economic analytics^25^— continue to drive traffic growth, while SDN-based infrastructures introduce new security challenges that demand resilient optical transport^26^. To the best of our knowledge, no prior work has jointly integrated mixed channel traffic grooming, shared backup path protection and XT-aware optical-layer provisioning within a unified IP over MCF-EON framework.

Joint optimisation of these three aspects is essential because they are tightly coupled. Grooming decisions determine the number of lightpaths established, which directly shapes the XT landscape: fewer lightpaths mean fewer adjacent-core contentions and lower aggregate XT. Conversely, XT constraints limit the set of feasible core-spectrum assignments and thereby restrict grooming opportunities, because a virtual link can only be created or extended when an XT-feasible lightpath exists. Similarly, SBPP’s capacity-sharing efficiency depends on XT-feasible core-spectrum assignments that are influenced by grooming decisions: aggressive grooming consolidates protection flows onto fewer lightpaths, amplifying the sharing benefit of SBPP, but only if those consolidated lightpaths satisfy XT constraints. Treating any one aspect in isolation therefore leads to suboptimal or even infeasible solutions.

Here we investigate mixed channel traffic grooming (MCTG) for SBPP-based IP over MCF-EONs, in which working and protection IP flows can be aggregated onto common optical channels under strict XT constraints. We develop a double auxiliary-graph routing, core and spectrum assignment framework (Double-AG-RCSA) that coordinates IP-layer grooming with crosstalk aware optical-layer resource allocation. One auxiliary graph exploits residual capacity of existing virtual links in the IP layer; the other performs XT-aware routing, core and spectrum assignment across feasible spectrum windows in the optical layer. Relative to our earlier study on mixed channel grooming for single-core IP-over-EONs^27^, the present work explicitly treats multicore fibres and inter-core crosstalk; relative to our previous study on crosstalk aware SBPP in MCF-EONs^19^, it introduces IP-layer grooming so that grooming and XT-aware provisioning are jointly optimised rather than treated separately. A preliminary version appeared as a conference paper^28^; the present manuscript substantially expands the formulation, introduces an explicit joint working–protection provisioning procedure, broadens the baseline comparisons, and evaluates performance under both static and dynamic traffic.

Three contributions follow. First, we extend mixed channel traffic grooming from single-core IP-over-EON settings to survivable IP over MCF-EONs with explicit XT constraints, enabling working and protection IP flows to share optical channels under SBPP. Second, we develop a double auxiliary-graph framework that jointly coordinates IP-layer grooming and XT-aware optical-layer provisioning, capturing the interaction between residual virtual-link capacity and crosstalk-constrained lightpath establishment. Third, we evaluate the framework comprehensively on the COST239 and NSFNET topologies under static and dynamic traffic, showing improved spectrum efficiency, crosstalk performance, protection-resource utilisation and blocking behaviour relative to representative baselines.

## Results

**Fig. 1:**
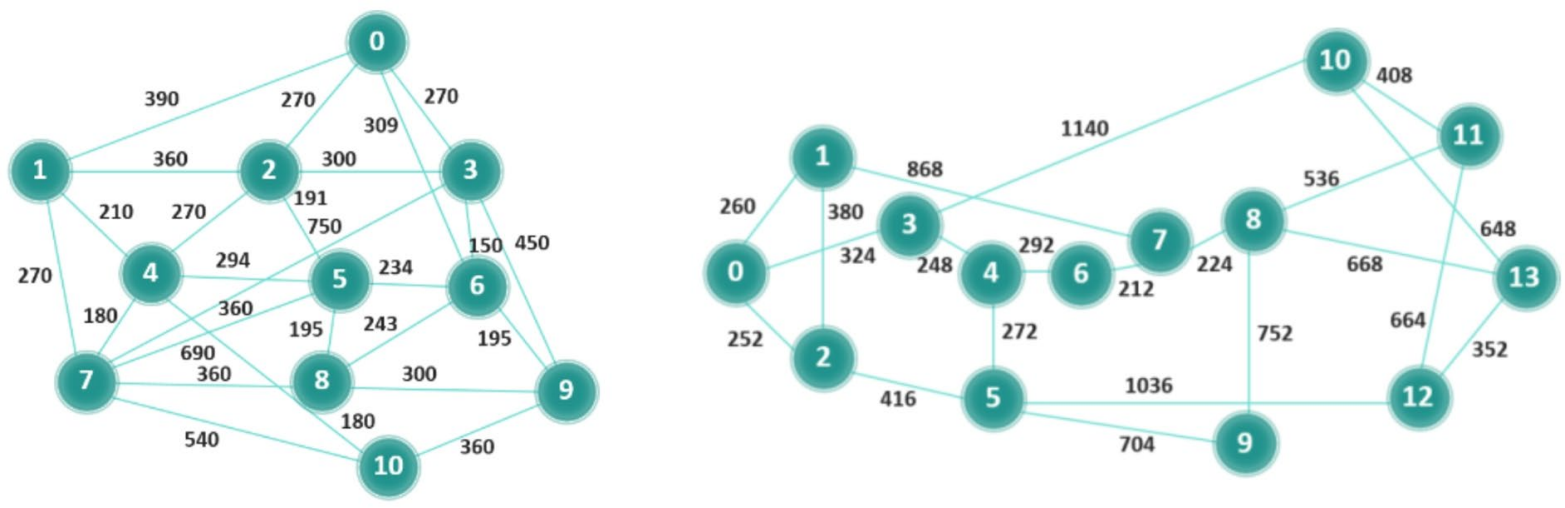
Test network topologies — combine (**a**) 11-node, 26-link COST239 and (**b**) 14-node, 21-link NSFNET into a single two-panel figure. Link lengths labelled in km.

We evaluated the Double-AG-RCSA framework on two backbone topologies: the 11-node, 26-link COST239 network with a 7-core MCF, and the 14-node, 21-link NSFNET network with a 19-core MCF (Fig. 1). Crosstalk was estimated using the parameters in Table 1 and a threshold of − 30 dB^29^; each frequency slot has a granularity of 12.5 GHz; BPSK, QPSK and 8-QAM modulation formats were considered with capacities and transparent reaches listed in Table 2. Traffic demands between node pairs followed a uniform distribution between 5 Gb/s and a maximum bandwidth X Gb/s, and each traffic matrix was evaluated under 1000 random permutations of request arrival order to mitigate ordering effects. Full simulation procedures and baseline definitions are provided in the Methods.

To isolate the contributions of mixed channel grooming, XT-aware provisioning, spectrum-window selection, and shared backup protection, we compared the proposed MixedTG-SBPP-XA-LC scheme with five reference schemes (Table 3): MixedTG-SBPP-XA-FF (first-fit spectrum selection), MixedTG-SBPP-XU (no XT awareness), SepTG-SBPP-XA-LC (separate-channel grooming), NoTG-SBPP-XA-LC (no grooming), and MixedTG-1 + 1-XA-LC (dedicated 1 + 1 protection).

### Crosstalk performance

**Fig. 2:**
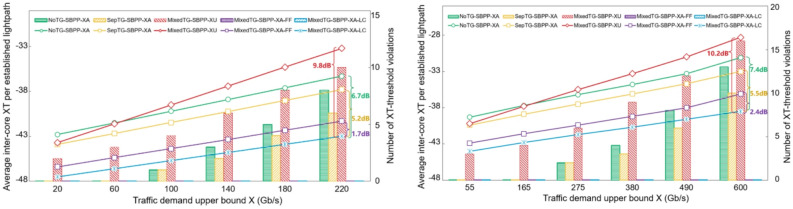
XT performance comparison — two-panel figure: (**a**) COST239 with 7-core MCF; (**b**) NSFNET with 19-core MCF. Bars: average XT per lightpath. Annotate XT-threshold violations under high load.

Inter-core crosstalk performance was quantified by the average XT per established lightpath and by the number of XT-threshold violations (cases in which predicted XT exceeds − 30 dB). Figure 2 summarises the results for the two topologies. The proposed MixedTG-SBPP-XA-LC scheme achieved the lowest average XT in both COST239-7-core and NSFNET-19-core, reducing the average XT by 9.8 dB and 10.2 dB respectively relative to the XT-unaware MixedTG-SBPP-XU scheme. Under high traffic load, MixedTG-SBPP-XU produced 10 XT violations in COST239-7-core and 16 violations in NSFNET-19-core, whereas both XT-aware schemes (XA-LC and XA-FF) produced none.

Among the two XT-aware spectrum-selection strategies, least-cost (LC) consistently outperformed first-fit (FF), with additional XT reductions of 1.7 dB and 2.4 dB in COST239-7-core and NSFNET-19-core respectively. The advantage was larger in the 19-core case, where higher core density leads to stronger inter-core coupling. Traffic grooming also influenced XT indirectly: SepTG-SBPP-XA-LC and NoTG-SBPP-XA-LC increased the average XT by about 5.2 dB and 6.7 dB in COST239-7-core, with 6 and 8 XT-threshold violations respectively; in NSFNET-19-core the increases reached 5.5 dB and 7.4 dB, with 10 and 13 violations. SepTG requires separate working and protection channels and NoTG disables aggregation, so both need more lightpaths, increase spectral fragmentation, and reduce opportunities to use low-XT cores.

### Spectrum efficiency and frequency-slot usage

**Fig. 3:**
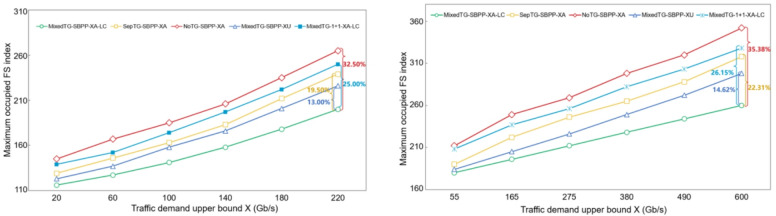
Maximum frequency-slot index — two-panel figure: (**a**) COST239 with 7-core MCF; (**b**) NSFNET with 19-core MCF.

Spectrum efficiency was measured by the maximum index of occupied frequency slots under static traffic (Fig. 3). MixedTG-SBPP-XA-LC consistently achieved the lowest usage in both networks. In COST239-7-core, mixed channel grooming reduced the maximum frequency-slot index by up to 19.5% relative to SepTG-SBPP-XA-LC and up to 32.5% relative to NoTG-SBPP-XA-LC; the corresponding reductions in NSFNET-19-core reached 22.3% and 35.4%. Compared with MixedTG-SBPP-XA-LC, the XT-unaware MixedTG-SBPP-XU scheme required 13.0% more spectrum in COST239-7-core and 14.6% more in NSFNET-19-core at high load, because XT-unaware provisioning selects routes and spectrum windows that can produce infeasible assignments, fragmentation and additional lightpath establishment. Protection strategy also had a significant effect: MixedTG-1 + 1-XA-LC increased the maximum frequency-slot index by 25.0% in COST239-7-core and 26.2% in NSFNET-19-core relative to SBPP, because 1 + 1 reserves a dedicated backup for each working lightpath and does not allow sharing.

### Protection-capacity sharing and backup efficiency

**Fig. 4:**
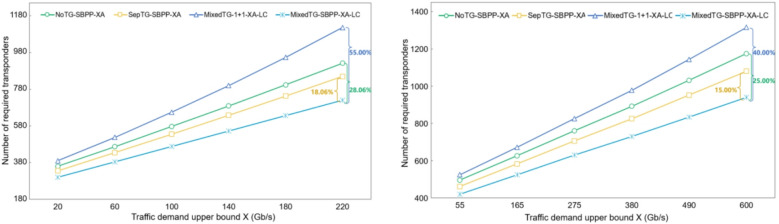
Number of used transponders — two-panel figure: (**a**) COST239 with 7-core MCF; (**b**) NSFNET with 19-core MCF.

Backup efficiency was assessed using a bandwidth-variable transponder model in which each lightpath occupies one transponder pair on up to 16 contiguous frequency slots on a single core (Fig. 4). Across both networks, SBPP substantially reduced the number of required transponders relative to 1 + 1 protection: MixedTG-SBPP-XA-LC reduced transponder count by up to 35.5% in COST239-7-core and 28.6% in NSFNET-19-core relative to MixedTG-1 + 1-XA-LC. Because the two schemes use the same grooming and XT-aware allocation, these differences are attributable solely to the protection mechanism. Among SBPP schemes, SepTG-SBPP-XA-LC and NoTG-SBPP-XA-LC required about 18 and 28% more transponders respectively than MixedTG-SBPP-XA-LC in COST239-7-core under high load, and about 15% and 25% more in NSFNET-19-core, reflecting the reduced grooming flexibility when working and protection traffic cannot share lightpaths.

## Discussion

The experiments show that joint coordination of mixed channel grooming, crosstalk aware provisioning and shared backup path protection yields consistent improvements across three largely orthogonal performance axes—spectrum efficiency, signal quality and backup-resource utilisation—without requiring trade-offs between them. The proposed Double-AG-RCSA framework delivered the lowest maximum frequency-slot index, the lowest average inter-core crosstalk and the lowest transponder count of all schemes compared, while eliminating crosstalk-threshold violations in both the 7-core and 19-core topologies.

The five baselines are designed as controlled experiments: each differs from the proposed MixedTG-SBPP-XA-LC scheme in exactly one factor (Table 3), so that performance differences can be attributed to the specific factor being varied rather than to confounding interactions. The comparisons isolate four technical factors. The contrast between MixedTG-SBPP-XA-LC and MixedTG-SBPP-XU indicates that XT awareness is necessary to ensure feasible RCSA decisions in MCF networks: without it, provisioning can produce infeasible assignments and systematic threshold violations. The contrast between least-cost (LC) and first-fit (FF) spectrum selection shows that spectrum-window selection rules have a measurable effect on XT, amplified in high-density fibres such as 19-core MCFs. The underlying mechanism is that FF selects the first feasible spectrum window in index order without considering the XT landscape, which may place lightpaths on cores that are already heavily loaded, accumulating unnecessary XT. LC, by contrast, evaluates all feasible windows via AG_o and selects the assignment that minimises incremental XT, thereby distributing lightpaths more evenly across the core-spectrum space and avoiding XT-dense regions. The contrast between mixed channel and separate-channel grooming (or no grooming) shows that aggregating working and protection flows onto shared lightpaths reduces the number of established lightpaths, which in turn reduces both spectrum occupation and the exposure of new channels to crosstalk. The contrast between SBPP and 1 + 1 protection confirms that sharing backup capacity among link-disjoint working paths substantially improves transponder efficiency at high load. The mechanism is that under 1 + 1 dedicated protection, each demand reserves a dedicated backup lightpath whose spectrum resources cannot be shared even when the corresponding working paths are failure-disjoint. SBPP relaxes this constraint by allowing backup resources to be shared among protection flows whose working paths do not share any common link, thereby significantly reducing total spectrum and transponder consumption. This sharing effect is amplified by mixed channel grooming, which further consolidates protection flows onto fewer lightpaths.

Our results also reveal an interaction between grooming and crosstalk that has not been quantified explicitly in previous work. Grooming affects crosstalk indirectly, by reducing the number of lightpaths that must be established and thus the opportunities for adjacent-core contention; conversely, crosstalk aware provisioning enables grooming by keeping residual virtual-link capacity high enough to absorb subsequent demands. Capturing this coupling is the primary motivation for the double auxiliary-graph design: the IP-layer auxiliary graph exposes residual-capacity reuse opportunities, while the optical-layer auxiliary graph performs XT-feasibility checks across candidate cores and spectrum windows. The two-stage, working-then-protection decomposition preserves tractability while keeping the cross-layer interaction visible to the solver.

Several limitations qualify these results. The evaluation is simulation-based and uses two specific topology and parameter settings; generalisation to other topologies, fibre types and load regimes requires further study. The crosstalk model follows the standard weak-coupling formulation of^4^ and does not include nonlinear or polarisation-mode effects that may become relevant in long-haul dense-SDM deployments. The current provisioning procedure is static and is evaluated under offline permutations of arrival orders; in production networks, arrival and teardown dynamics would interact with grooming decisions in ways that our preliminary dynamic-traffic experiments only partially capture. Future work will extend the framework to dynamic traffic at larger scale, incorporate richer physical-layer impairments, and examine implementation-oriented validation in control-plane testbeds.

Overall, the findings indicate that mixed channel grooming, XT-aware provisioning and shared backup protection are most effective when designed and optimised together rather than layered sequentially. The Double-AG-RCSA framework offers a concrete and extensible route to this joint optimisation for survivable IP over MCF-EONs.

## Methods

### Network architecture

We consider an IP over MCF-EON modelled as a directed graph G = (N, L), where N denotes the set of nodes and L the set of physical links. Each physical link l ∈ L carries an MCF with C cores, and each core supports F frequency slots of bandwidth 12.5 GHz each; the available spectrum on each core is indexed by f ∈ {1, 2, …, F}. A connection request (IP flow) is represented as d_k = (s_k, t_k, b_k), where s_k and t_k denote the source and destination nodes and b_k the required bandwidth. Depending on the modulation format (BPSK, QPSK or 8-QAM), each request occupies a number of consecutive frequency slots and is subject to a maximum transparent reach R_m. The frequency-slot demand of flow k is denoted W_k.

### Crosstalk estimation

Inter-core crosstalk originates from power coupling between adjacent cores and accumulates with transmission distance. Following the coupling model of^4^, the crosstalk between cores i and j on link l with length $\:Le{n}_{l}$ is

1 $$\:X{T}_{i,j}^{l}=\mathrm{t}\mathrm{a}\mathrm{n}\mathrm{h}({h}_{i,j} \cdot Le{n}_{l})$$

where h_{i, j} is the power coupling coefficient between cores i and j, given by

2 $$\:{h}_{i,j}=2 \cdot {k}_{i,j}^{2} \cdot {b}_{r}/(\beta\: \cdot {{\Lambda\:}}_{i,j})$$

where b_r is the bending radius, β the propagation constant, k_{i, j} the mode coupling coefficient and Λ_{i, j} the core pitch. The mode coupling coefficient is

3 $$\kappa _{{i,j}} = \left( {\Delta \cdot cr\cdot U^{2} } \right)/(V^{3} \cdot K_{0} \left( { \wedge _{{i,j}} /cr\cdot W} \right)\cdot K_{1} \left( {W)^{2} } \right)$$

where Δ is the relative refractive-index difference, cr the core radius, U and W the normalised transverse wavenumbers in core and cladding, and V the normalised frequency. For a lightpath p traversing multiple links, the total accumulated crosstalk is

4 $$\:X{T}_{p}={\varSigma\:}_{l\in\:p}{\varSigma\:}_{i,j\in\:C}X{T}_{i,j}^{l}$$

and must satisfy the crosstalk constraint

5 $$\:X{T}_{p}\le\:X{T}_{t}h$$

where XT_th is the crosstalk threshold, typically set to − 30 dB to guarantee acceptable quality of transmission. During core and frequency-slot assignment, a candidate core is considered unavailable if the estimated crosstalk between an existing lightpath and the candidate lightpath would exceed XT_th.

### Mixed channel traffic grooming with SBPP

**Fig. 5:**
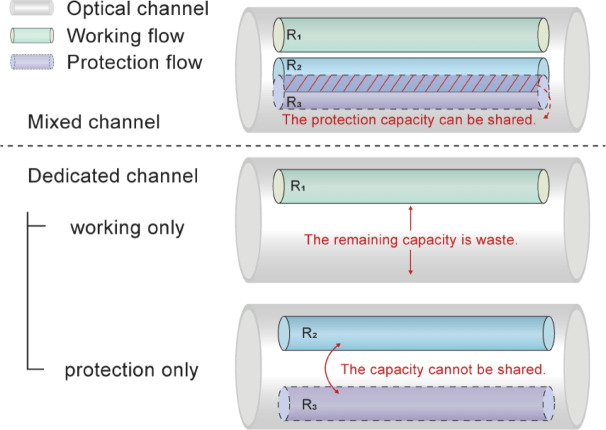
Mixed- and dedicated-channel traffic-grooming configurations between nodes S and D under SBPP.

The proposed framework integrates SBPP for survivability with traffic grooming for resource efficiency. In an IP over MCF-EON, traffic grooming aggregates multiple IP flows onto lightpaths (optical channels) established between node pairs. Mixed channel traffic grooming (MCTG) extends this principle by allowing a single lightpath to simultaneously carry both working and protection flows when SBPP constraints are satisfied^28^. In the mixed channel mode illustrated in Fig. 5, a working flow R1 and protection flows R2 and R3 can be aggregated onto the same optical channel provided that the working paths associated with R2 and R3 are link-disjoint from the optical path of R1; this disjointness guarantees that a failure affecting the working path of R1 does not simultaneously compromise the protection paths. The dedicated-channel mode restricts each lightpath to one flow type; working channels then leave capacity stranded (unused bandwidth cannot be reallocated to protection), and protection-dedicated lightpaths cannot share backup capacity beyond simple co-location. Mixed channel grooming therefore achieves a better balance between spectral efficiency and survivability.

### Double-AG-RCSA framework

The objective is to minimise spectrum usage and inter-core crosstalk while provisioning both working and protection IP flows under SBPP. We assume that each lightpath occupies a contiguous set of frequency slots on a single core along its entire route; that spectrum continuity and non-overlap are enforced on all traversed links; that each lightpath uses a single modulation format; and that crosstalk accumulates additively on a dB scale along the path. Under these assumptions we develop the double auxiliary-graph routing, core and spectrum assignment framework (Double-AG-RCSA), combining two reusable modules: an IP-layer auxiliary graph AG_i that tests whether a flow can be accommodated through grooming over the current virtual topology, and an optical-layer auxiliary graph AG_o that establishes a new lightpath through XT-aware routing, core selection and spectrum assignment when grooming is infeasible.

**Fig. 6:**
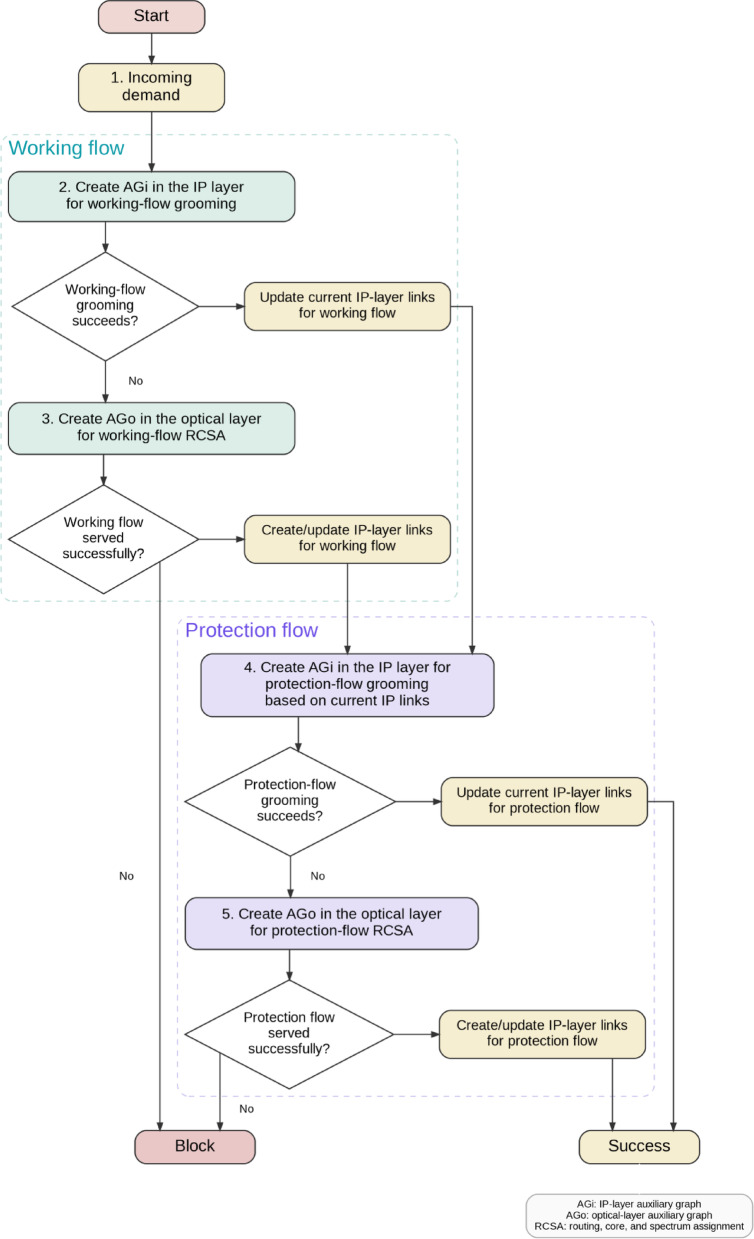
Flowchart of the double-AG-RCSA algorithm.

Each incoming demand is provisioned in two successive stages: working-flow provisioning followed by protection-flow provisioning (Fig. 6). To clarify the interaction between AG_i and AG_o, we trace the complete lifecycle of a single demand $\:{d}_{k}=({s}_{k},{t}_{k},{b}_{k})$ from arrival to final disposition. Step 1 (IP-layer grooming attempt for working flow): the algorithm constructs AG_i from the current virtual topology G_v and searches for a least-cost path with sufficient residual capacity. If such a path exists, the working flow is groomed onto existing lightpaths and the residual capacities of the traversed IP links in G_v are updated. Step 2 (Optical-layer provisioning for working flow): if AG_i yields no feasible path, the algorithm computes K-shortest physical routes, selects a modulation format based on the route length, and constructs AG_o for each candidate spectrum window. The core-spectrum assignment with the lowest XT cost that satisfies the XT constraint is selected, a new lightpath is established, and G_v is updated with the new IP link. Step 3 (Protection-flow provisioning): after the working flow is established, the same two-stage procedure (AG_i then AG_o) is applied for the protection flow on the updated G_v, with the additional SBPP constraint that backup capacity on a link can be shared only among protection flows whose corresponding working paths are link-disjoint. During AG_o construction for protection, core eligibility is further restricted: working-occupied cores are excluded, and protection-occupied cores are eligible only under the link-disjointness condition (Fig. 5). Step 4 (Blocking): if neither IP-layer grooming nor optical-layer provisioning succeeds for either the working or protection flow, the demand is blocked and the network state is unchanged.

### IP-layer virtual topology and construction of AG_i

The Double-AG-RCSA framework maintains an IP-layer virtual topology G_v consisting of logical IP links between node pairs, each supported by one or more previously established optical lightpaths and characterised by its residual capacity (Fig. 6). G_v initially contains all IP-layer nodes but no IP links; as working or protection lightpaths are established, corresponding IP links are created or updated. For example, a working lightpath on A–B at 8-QAM occupying four frequency slots carries 300 Gb/s; after 260 Gb/s are loaded, 40 Gb/s residual capacity remains for subsequent grooming.

**Fig. 7:**
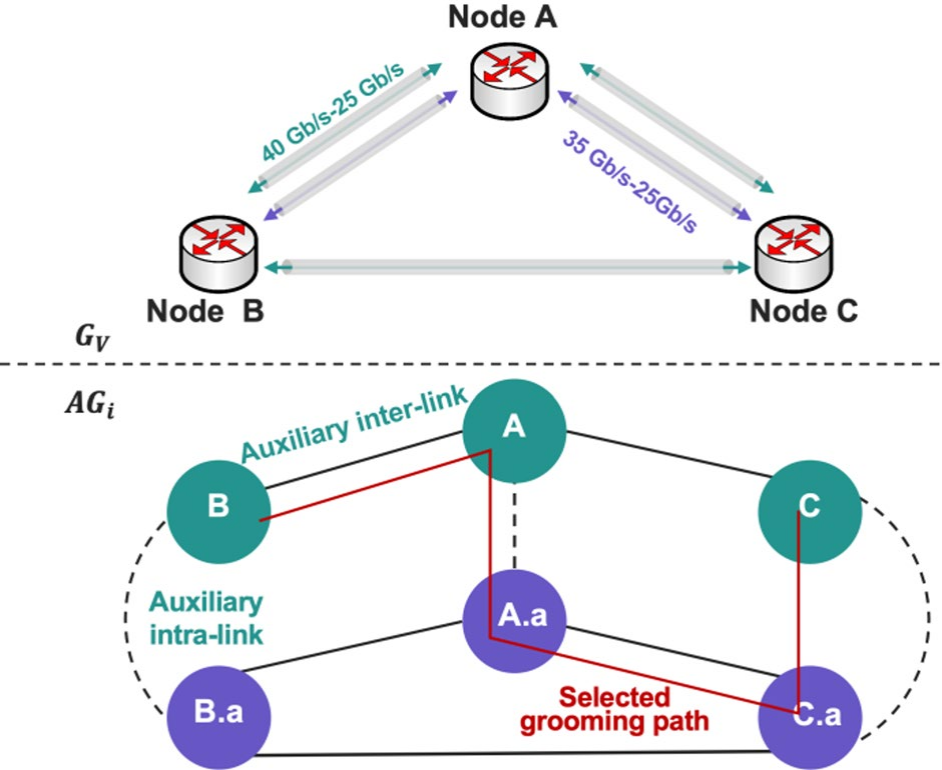
Construction of the IP-layer auxiliary graph AG_i from the current virtual topology.

AG_i is constructed from G_v as follows (Fig. 7). Each original IP-layer node is associated with an auxiliary counterpart; zero-cost intra-links connect each node to its auxiliary counterpart (e.g., A–A.a); and auxiliary inter-links between auxiliary nodes represent all available IP links between node pairs, each annotated with residual capacity. A standard shortest-path algorithm applied to AG_i determines whether the current flow can be accommodated through grooming. In the worked example, a grooming path B → A → A.a → C.a → C shows that a 25 Gb/s B–C demand is served by reusing an existing A–B working IP link and an A–C protection IP link; residual capacities are updated accordingly. If no feasible path exists, the flow is forwarded to AG_o.

### Optical-layer auxiliary graph AG_o

When grooming is infeasible, the framework performs optical-layer provisioning. After identifying a candidate physical route and selecting an appropriate modulation format, the required number of frequency slots is determined according to the bandwidth demand. To satisfy the spectrum contiguity and continuity constraints in elastic optical networks, the concept of a spectrum window (SW) is adopted. A spectrum window is defined as a contiguous block of frequency slots on the same core, with a size no smaller than the number of slots required by the demand under the selected modulation format. A spectrum window is considered available only if all constituent slots are free, and the same SW must be available on every link along the candidate physical route to satisfy spectrum continuity.

For each feasible spectrum window, a separate instance of AG_o is constructed to perform XT-aware routing, core selection and spectrum assignment. Figure 4 illustrates an example in which the optical route A–B–C consists of 3-core MCF links and a spectrum window spanning slots 1–4 is examined for provisioning a new lightpath. Each physical node X is expanded into two groups of auxiliary nodes: a left group representing ingress access to each core and a right group representing egress access from each core. The source is represented by a single left-side node and a full right-side group; the destination by a full left-side group and a single right-side node. Cores that cannot support the selected spectrum window (due to prior slot occupancy) are marked unavailable and are not connected by auxiliary links. Each left-side auxiliary node is connected to its corresponding right-side auxiliary node by an intra-link whose cost reflects the current utilisation state of the destination core: unused cores receive a high intra-link cost (e.g., 1000) to discourage opening a new core, whereas partially occupied cores receive a low cost (e.g., 0.001) to promote continuity and reuse.

**Fig. 8:**
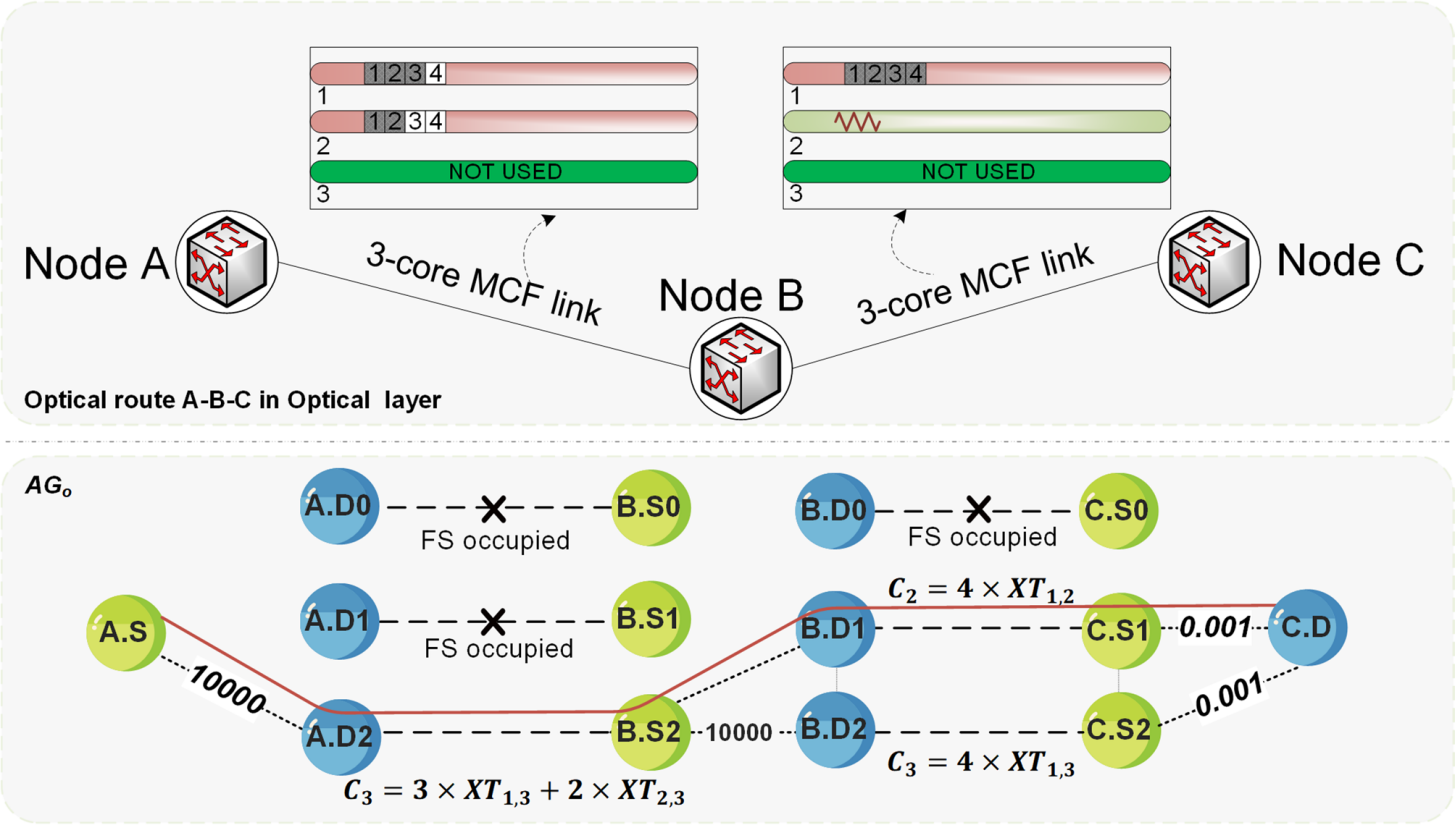
Construction of the optical-layer auxiliary graph AG_o for a candidate route and spectrum window.

Inter-links between auxiliary nodes of adjacent physical nodes represent feasible provisioning of the selected spectrum window over each MCF link. For each slot-available core on link X–Y, an inter-link is added with a cost reflecting the estimated XT incurred when the new lightpath is assigned to that core. If core 3 is selected and cores 1 and 2 already carry traffic on overlapping slots, the XT cost is computed as the sum of XT contributions from adjacent occupied cores over all overlapping slots. A depth-first search then enumerates candidate routes through AG_o in ascending order of total cost; for each candidate, XT feasibility is verified for every slot in the selected spectrum window, and the XT values of overlapping existing lightpaths are recalculated. A route is accepted only if all new and existing lightpaths remain XT-feasible. In the worked example (Fig. 8), the procedure selects route A–B–C using core 3 on link A–B and core 2 on link B–C, with slots 1–4 assigned.

**Fig. 9:**
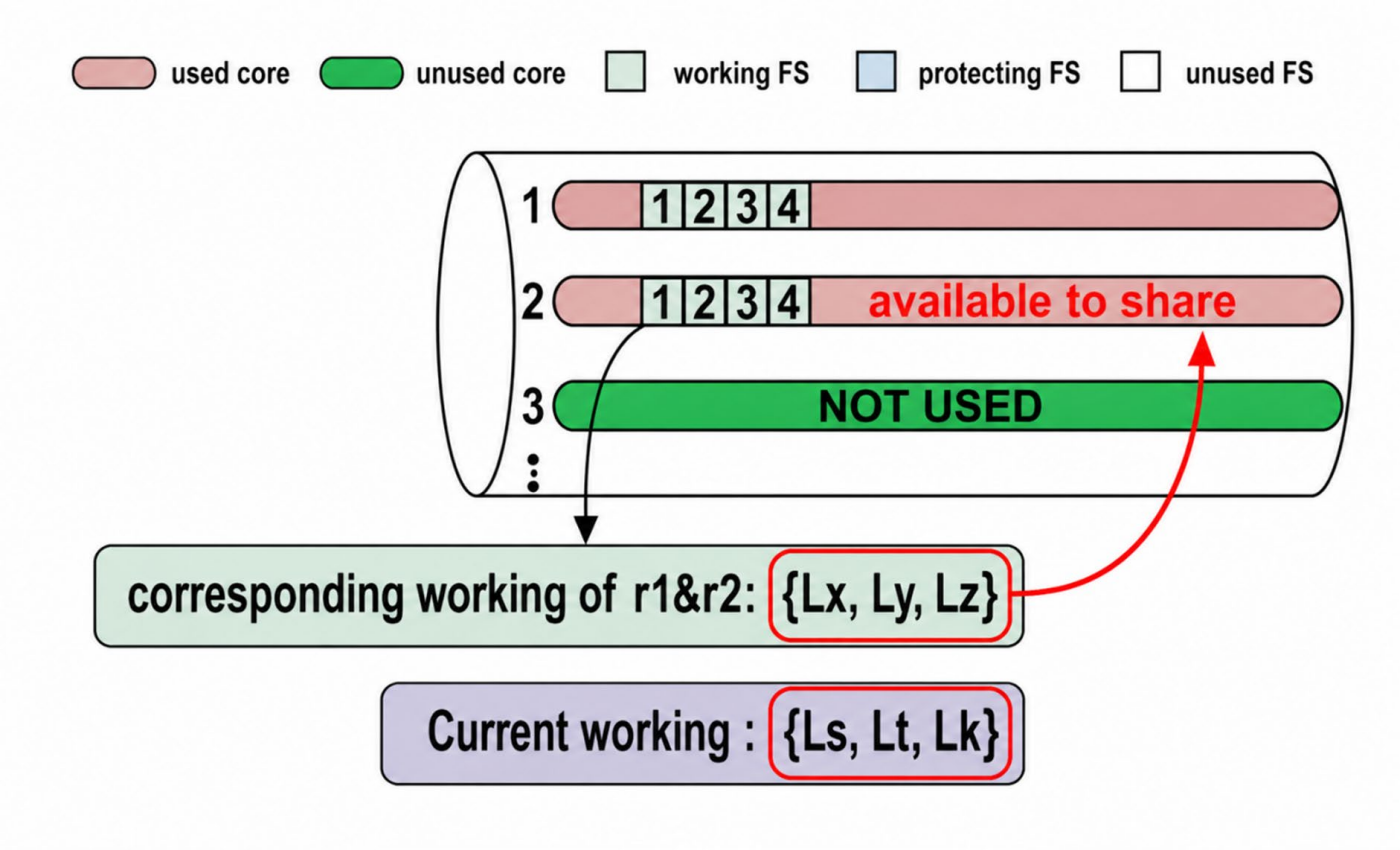
Core-eligibility evaluation for protection provisioning in the optical layer under SBPP.

Once the working lightpath is established, G_v is updated and the protection flow is provisioned on the updated virtual topology. The protection flow is first attempted through AG_i with SBPP-specific sharing constraints (a protection IP link can be reused only when the associated working path is link-disjoint from the working path of the current request). If grooming fails, AG_o is invoked and a link-disjoint protection path must be found. During construction of AG_o for a protection flow, SBPP constraints restrict the cores eligible for inter-link representation (Fig. 9): an unused core is always eligible; a core occupied by a working flow is excluded, since working capacity cannot be shared; a core already used by protection flows is eligible only if the corresponding working paths are link-disjoint from the working path of the current request. For each eligible core, an inter-link is added with a cost that reflects both the XT introduced by placing the protection lightpath on that core and a consolidation term that promotes protection-resource sharing. Candidate protection routes undergo the same XT-feasibility verification as working routes. When an XT-feasible, link-disjoint protection route is found, the working and protection lightpaths are committed jointly and the network state (slot usage, G_v, XT tables) is updated.

### Complexity analysis

The computational complexity of Double-AG-RCSA is determined by the two auxiliary-graph procedures and the SBPP feasibility checks in each layer. IP-layer grooming uses shortest-path search on AG_i with complexity O(V^2), where V is the number of IP-layer nodes. Constructing AG_o for a single spectrum window requires evaluating frequency-slot availability and XT contributions for all cores on each link of the candidate physical route, which costs O(|L| · C · W), where |L| is the number of links on the route, C the number of cores per link and W the number of frequency slots in the spectrum window. For K incoming demands, the total complexity is O(K · (V^2 + |L| · C · W)).

### Simulation setup and baselines

Simulations were conducted on the 11-node, 26-link COST239 network with a 7-core MCF and on the 14-node, 21-link NSFNET network with a 19-core MCF (Fig. 1; link lengths in kilometres are labelled). Cross-sections of the two multicore fibres are provided as Supplementary Fig. S1. Crosstalk parameters (Table 1) follow^12^, and the threshold was set to − 30 dB^29^ uniformly across modulation formats. Each frequency slot has a granularity of 12.5 GHz; BPSK, QPSK and 8-QAM were considered, with capacities and transparent reaches as in Table 2. A one-slot guard band was reserved between adjacent lightpaths. For each source–destination pair, K = 3 shortest paths were precomputed using Yen’s algorithm as candidate physical routes. For the spectrum-window traversal, the algorithm scans contiguous spectrum windows from the lowest-indexed frequency slot upward; under the first-fit (FF) strategy the first feasible window is selected, whereas under the least-cost (LC) strategy all feasible windows are evaluated via AG_o and the one with the lowest total XT cost is chosen. The cost assigned to each inter-link in AG_o equals the estimated incremental XT (in linear scale) that the candidate core-spectrum assignment would add to existing lightpaths on that link; no additional weighting factors are applied, so the objective is pure XT minimisation among feasible assignments. Traffic demands between node pairs followed a uniform distribution between 5 Gb/s and a maximum bandwidth X Gb/s. For the static-traffic evaluation, each traffic matrix was evaluated under 1000 random permutations of request arrival order; selecting a single best ordering tightened the upper bound by about 3% in preliminary tests but did not alter the relative ranking of the compared schemes. For the dynamic-traffic evaluation, connection requests arrive according to a Poisson process and holding times follow a negative exponential distribution; the offered load (in Erlangs) is varied by adjusting the mean inter-arrival time while keeping the mean holding time fixed, and the specific Erlang values tested are indicated on the x-axes of the corresponding figures. All experiments were repeated with 10 independent random seeds (seeds 1–10), and the reported results are averages over these 10 runs; error bars in the figures indicate 95% confidence intervals. Performance was evaluated by the maximum frequency-slot index, transponder usage, average crosstalk, and protection-capacity sharing.

**Table 1:** MCF parameters used for crosstalk calculation^12^.

Parameter	Value
Δ (relative refractive-index difference)	0.35%
cr (core radius)	3.8 μm
V (normalised frequency)	2.2
U	√(V² − W²)
W	1.1428·V − 0.996
br (bending radius)	10 cm
β (propagation constant)	4 × 10⁶ m⁻¹

**Table 2:** Frequency-slot capacity and transparent reach for the three modulation formats^27^.

Modulation format	FS capacity (Gb/s)	Transparent reach (km)
BPSK	25	4000
QPSK	50	2000
8-QAM	75	1000

Five baselines were used (Table 3). MixedTG-SBPP-XA-FF replaces least-cost with first-fit spectrum-window selection. MixedTG-SBPP-XU removes XT awareness. SepTG-SBPP-XA-LC uses separate-channel grooming. NoTG-SBPP-XA-LC disables grooming. MixedTG-1 + 1-XA-LC replaces SBPP with dedicated 1 + 1 protection. Comparisons target four technical factors: mixed channel versus separate-channel or no grooming; XT-aware versus XT-unaware provisioning; least-cost versus first-fit spectrum-window selection; and SBPP versus 1 + 1 protection.

**Table 3:** Summary of compared schemes.

Scheme	Grooming mode	XT awareness	Spectrum-window rule	Protection
MixedTG-SBPP-XA-LC	Mixed	Yes	Least-cost	SBPP
MixedTG-SBPP-XA-FF	Mixed	Yes	First-fit	SBPP
MixedTG-SBPP-XU	Mixed	No	—	SBPP
SepTG-SBPP-XA-LC	Separate	Yes	Least-cost	SBPP
NoTG-SBPP-XA-LC	None	Yes	Least-cost	SBPP
MixedTG-1 + 1-XA-LC	Mixed	Yes	Least-cost	1 + 1

### Use of large language models

No large language models were used to generate the scientific content, results or conclusions of this work. Large language models were used only for language editing of the manuscript; all technical content, experimental design, analysis and interpretation were produced by the authors.

## Supplementary Information

Below is the link to the electronic supplementary material.

Supplementary Material 1

## Data Availability

The simulation code and anonymised network and traffic data used to generate the results in this study are available from the corresponding author upon reasonable request. The COST239 and NSFNET topologies used in the experiments are publicly available in the literature cited in this manuscript.

## References

[1] Gerstel, O., Jinno, M., Lord, A. & Ben Yoo, S. J. Elastic optical networking: a new dawn for the optical layer? *IEEE Commun. Mag*. **50**, s12–s20 (2012).

[2] Winzer, P. J. Spatial multiplexing: the next frontier in network capacity scaling. In *Proc. ECOC* (2013).

[3] Saridis, G. M., Alexandropoulos, D., Zervas, G. & Simeonidou, D. Survey and evaluation of space division multiplexing: from technologies to optical networks. *IEEE Commun. Surv. Tutor.* **17**, 2136–2156 (2015).

[4] Mizuno, T., Takara, H., Sano, A. & Miyamoto, Y. Dense space-division multiplexed transmission systems using multi-core and multi-mode fibre. *J. Lightw Technol.* **34**, 582–592 (2015).

[5] Zhu, K. & Mukherjee, B. Traffic grooming in WDM optical networks: past, present, and future. *IEEE Netw.* **16**, 46–56 (2002).

[6] Zang, H., Jue, J. P. & Mukherjee, B. A review of traffic grooming in WDM optical networks: architectures and challenges. *Opt. Netw. Mag*. **4**, 55–64 (2003).

[7] Zhu, R. et al. Crosstalk-aware RCSA for spatial-division multiplexing enabled elastic optical networks with multi-core fibres. *J. Lightw Technol.* **34**, 4068–4079 (2016).

[8] Klinkowski, M. & Zalewski, G. Dynamic crosstalk-aware lightpath provisioning in spectrally-spatially flexible optical networks. *J. Opt. Commun. Netw.* **11**, 213–225 (2019).

[9] Klinkowski, M., Lechowicz, P. & Walkowiak, K. Survey of resource-allocation schemes and algorithms in spectrally-spatially flexible optical networking. *Opt. Switch Netw.* **27**, 58–78 (2018).

[10] Zhu, K. & Mukherjee, B. A review of survivability mechanisms in optical networks. *Opt. Netw. Mag*. **4**, 16–23 (2003).

[11] Guo, H. et al. Shared-backup path protection in flexible-grid optical networks. *J. Lightw Technol.* **33**, 3709–3720 (2015).

[12] Tang, F., Yan, Y., Peng, L., Bose, S. K. & Shen, G. Crosstalk-aware counter-propagating core assignment to reduce inter-core crosstalk and capacity wastage in MCF optical networks. *J. Lightw Technol.* **37**, 5010–5027 (2019).

[13] Pinto-Ríos, J. et al. Resource allocation in multicore elastic optical networks: a deep reinforcement learning approach. *J. Opt. Commun. Netw.* **15**, A60–A72 (2023).

[14] Chen, Y. et al. Crosstalk classification based on synthetically considering crosstalk and fragmentation in SDM-EONs. *Photonics* **10**, 340 (2023).

[15] Zhang, J. et al. Dynamic multi-layer traffic grooming in elastic optical networks. *J. Lightw Technol.* **38**, 4169–4181 (2020).

[16] Velasco, L. et al. Cross-layer orchestration of IP/optical networks with traffic grooming. *J. Opt. Commun. Netw.* **14**, B46–B58 (2022).

[17] Wang, H. et al. Energy-efficient and delay-aware traffic grooming in elastic optical networks. *Opt. Switch Netw.* **46**, 100623 (2022).

[18] Li, X. et al. Learning-assisted dynamic traffic grooming in flexible-grid optical networks. *J. Opt. Commun. Netw.* **15**, A120–A132 (2023).

[19] Tang, F., Shen, G. & Rouskas, G. N. Crosstalk-aware shared backup path protection in multi-core fibre elastic optical networks. *J. Lightw Technol.* **39**, 3025–3036 (2021).

[20] Moghaddam, E. E., Beyranvand, H. & Salehi, J. A. Crosstalk-aware resource allocation in survivable space-division-multiplexed elastic optical networks supporting hybrid dedicated and shared path protection. *J. Lightw Technol.* **38**, 1095–1102 (2020).

[21] Miyazawa, T. et al. Efficient shared path protection for survivable EONs. *J. Opt. Commun. Netw.* **11**, 441–450 (2019).

[22] Yin, S. et al. XT-considered multiple-backup-paths and resources-shared protection scheme based on ring covers. *Opt. Express*. **29**, 6737–6755 (2021). 33726188 10.1364/OE.400042

[23] Li, S. & Huang, S. Heterogeneous attention fusion network with multi-branch decoding for UAV semantic segmentation. *J. King Saud Univ. – Comput. Inf. Sci.*10.1007/s44443-026-00757 (2026).

[24] Zhu, B. & Niu, L. A privacy-preserving federated learning scheme with homomorphic encryption and edge computing. *Alex Eng. J.* **118**, 11–20. 10.1016/j.aej.2024.12.070 (2025).

[25] Liu, D., Shen, Q. & Liu, J. The health-wealth gradient in labor markets: Integrating health, insurance, and social metrics to predict employment density. *Computation* **14**, 22. 10.3390/computation14010022 (2026).

[26] Tang, D. et al. MWD-CFM: Detection and mitigation of DDoS attack against SDN flow tables. *IEEE Trans. Netw.* **34**, 4269–4282 (2026).

[27] Tang, F. et al. Mixed-channel traffic grooming for IP-over-EONs with SBPP-based cross-layer protection. *J. Lightw Technol.* **35**, 3836–3848 (2017).

[28] Tang, F., Wang, S. & Shen, G. Mixed-channel traffic grooming in an SBPP-based IP over MCF-EON with minimised inter-core crosstalk. In *Proc. ACP/POEM* (2023).

[29] Muhammad, A., Zervas, G., Simeonidou, D. & Forchheimer, R. Routing, spectrum and core allocation in flexgrid SDM networks with multi-core fibres. In *Proc. ONDM 2014* 192–197 (2014).

[30] Halder, J., Paira, S., Acharya, T. & Bhattacharya, U. Design of a novel XT-aware energy- and spectrum-efficient RSCA scheme in offline SDM-EON. Opt. *Fiber Technol.* **63**, 102502 (2021).

