# Supplementary material for: Mixed channel traffic grooming with crosstalk awareness and shared backup path protection in IP over multicore fibre elastic optical networks
Source: Sci Rep. 2026 Jun 5;16:25748. doi: 10.1038/s41598-026-55987-8 (PMC13482616; doi:10.1038/s41598-026-55987-8)
Supplement: Supplementary file 1 [file 41598_2026_55987_MOESM1_ESM.pdf]

Supplementary Fig. S1

Cross-sections of the multi-core fibres used in the simulations: (a) 7-core MCF and (b) 19-core MCF.

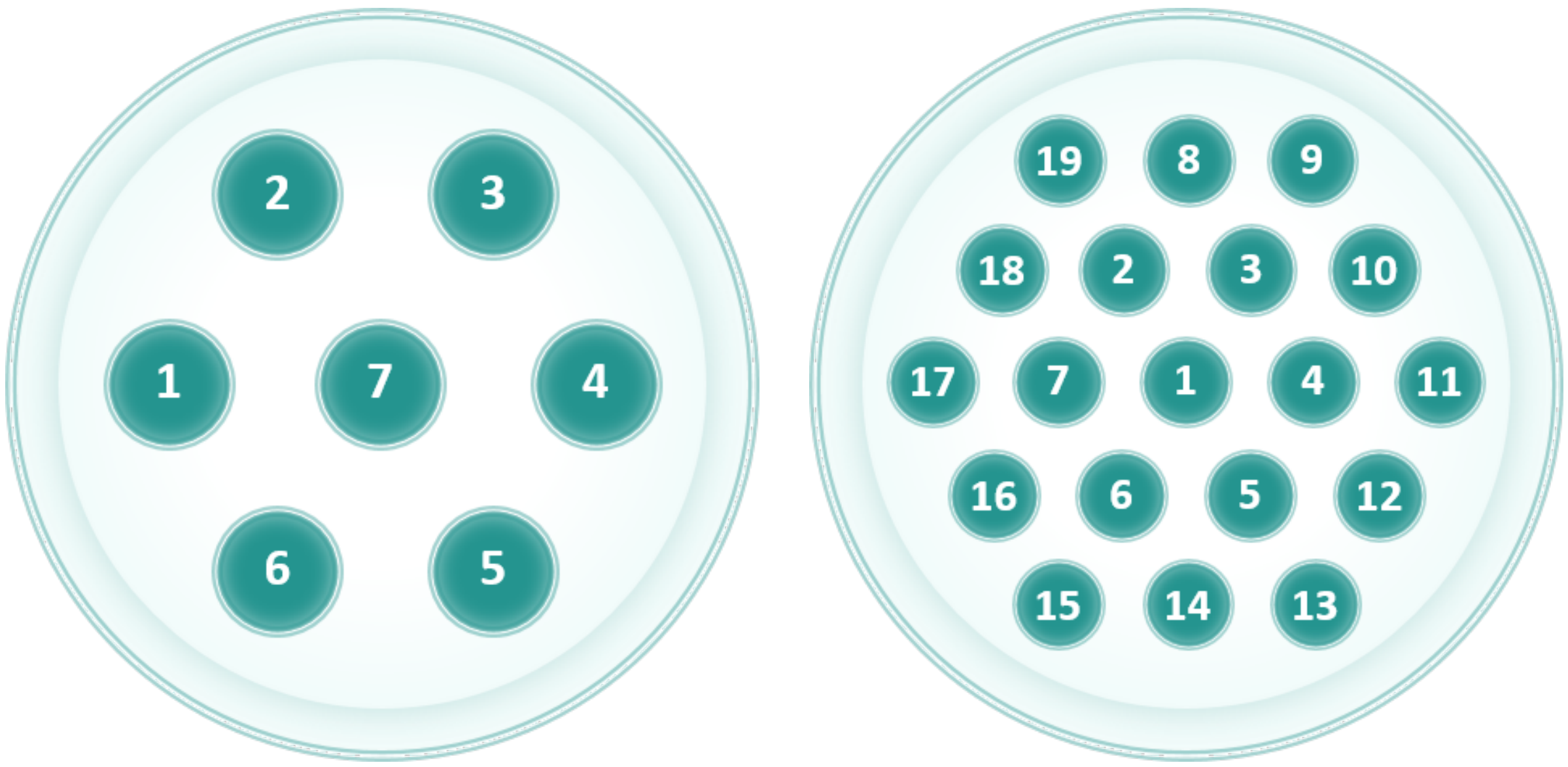

(a) 7-core MCF

(b) 19-core MCF
